# Gait coordination in overground walking with a virtual reality avatar

**DOI:** 10.1098/rsos.200622

**Published:** 2020-07-15

**Authors:** Artur A. Soczawa-Stronczyk, Mateusz Bocian

**Affiliations:** 1 School of Engineering, University of Leicester, Leicester, UK; 2 Biomechanics and Immersive Technology Laboratory, University of Leicester, Leicester, UK

**Keywords:** virtual reality, interpersonal coordination, stepping behaviour, walking avatars, gait biomechanics

## Abstract

Little information is currently available on interpersonal gait synchronization in overground walking. This is caused by difficulties in continuous gait monitoring over many steps while ensuring repeatability of experimental conditions. These challenges could be overcome by using immersive virtual reality (VR), assuming it offers ecological validity. To this end, this study provides some of the first evidence of gait coordination patterns for overground walking dyads in VR. Six subjects covered the total distance of 27 km while walking with a pacer. The pacer was either a real human subject or their anatomically and biomechanically representative VR avatar driven by an artificial intelligence algorithm. Side-by-side and front-to-back arrangements were tested without and with the instruction to synchronize steps. Little evidence of spontaneous gait coordination was found in both visual conditions, but persistent gait coordination patterns were found in the case of intentional synchronization. Front-to-back rather than side-by-side arrangement consistently yielded in the latter case higher mean synchronization strength index. Although the mean magnitude of synchronization strength index was overall comparable in both visual conditions when walking under the instruction to synchronize steps, quantitative and qualitative differences were found which might be associated with common limitations of VR solutions.

## 1. Introduction

Spontaneous gait coordination in walking has become one of the canonical examples of an emergent behaviour between autonomous agents [1–3]. Persistent gait coordination patterns have been reported in dyadic walking in side-by-side [4–13] and front-to-back [14,15] pedestrians’ arrangements. The gait coordination strength and directionality has been shown to strongly depend in this case on the type and the amount of available sensory information. However, the results from dyadic walking cannot be simply extrapolated to walking in groups and crowds—a situation commonly encountered in real-world settings, while data enabling gait coordination patterns to be examined in the latter case are still sparse [16–19].

Only a few studies investigated spontaneous gait synchronization in real-life environment [4,16,17,19]. The main reasons for this pertain to controllability of experimental conditions and observability of measured variables. On the one hand, the desire of closely controlled setting is difficult to realize in an environment subjected to various disturbances. On the other hand, the inference of spatial and temporal gait variables demands a distributed instrumentation system capable of simultaneous capture of data generated by multiple pedestrians. Consequently, alternative methods of investigating gait adaptations between pedestrians were proposed, predominantly relying on treadmills. However, the ecological validity of results from studies other than those enabling overground walking can be put in question [20,21].

Considering these limitations, virtual reality (VR) technology is thought to have the potential to boost the efficiency of research on locomotion. The current state of VR technology allows users to interact with near-realistic virtual environments, including autonomous agents. To this day, VR locomotion studies have been concerned with spatial cognition and awareness [22–24], locomotor training [25–33], locomotion and perception in real-world and virtual environment [34–39], navigation [40–42], collision avoidance [43,44] and crowd behaviour [45,46]. Locomotion interfaces allowing users to interact with and explore the VR content include a joystick [47], walking-in-place [48,49], treadmill [50–56] and those enabling overground walking compatible with real-world experience [35,36,57]. The last solution shows the highest promise in capturing pedestrians’ natural behaviour [40–42,49,58,59], assuming it employs isometric rather than non-isometric mapping. The former technique retains motion parameters such as translation and rotation, and their time derivatives, of those from the real environment in the virtualized equivalent, whereas the latter modifies some or all of these parameters [60]. (For a detailed review of developments on human locomotion in VR the reader is referred to Steinicke *et al.* [38].)

However, studies on the overground locomotion in VR, employing isometric mapping, are still rare and their main focus remains the gait kinematics and sensory integration rather than interaction with VR-based agents [35,57,61]. Furthermore, the gait enabled in these studies by VR interfaces can hardly be considered unconstrained, as the walkers are either accompanied by a man-handled trolley carrying PC provision or their locomotion is realized over few gait cycles.

It is important to note that VR comes with certain inherent limitations. Previous comparative studies on gait parameters during walking in real and virtual environment found underestimation of egocentric distances [62] as well as decrease of the walking speed and the stride length a common response to VR immersion [63]. This was suggested to be caused by the altered distance perception in virtual reality, often referred to as distance compression [36,64–67], which may become negligible after a 5 min period of habituation to virtual environment [68]. Some more recent studies found walking and the motion adaptation in VR to be quantitatively comparable to those in the real-world environment [36,43,44,69,70]. A widely acknowledged consensus is that while quantitative differences between virtual and real-life locomotion might still exist, the pedestrian behaviour in both environments is qualitatively compatible [34,43,44,71,72].

To address the gap in current knowledge, the ambition of this study is to develop a VR platform enabling unconstrained overground walking in the presence of virtual agents while preserving ecological validity with regards to the behaviour in real-life settings. The main advantages of this solution in human locomotion studies derive from controllability and observability [44,46], typically associated with testing in a laboratory environment, and the ability of testing complex, uncommon or even unrealistic scenarios [73]. To validate the platform, six subjects were asked to walk with a real and virtual pacer in various topological arrangements while having their gait monitored. Gait kinematics were analysed to identify any coordination patterns.

It was hypothesized that a VR locomotor interface enabling unconstrained overground walking can evoke gait coordination patterns between a real pedestrian and a virtual reality avatar similar to those observed when walking in the corresponding conditions in the real-life environment. Informed by the previous studies on walking in pairs [9,10] and groups [16–19], it was expected that the spontaneous synchronization of gait between walkers, if present, would be of transient nature [9,10]. Given a sufficiently long walking path is provided, it was expected that the spontaneous synchronization of gait would be relatively weak. Due to a lesser number of available synchronization stimuli when compared to walking in a group of pedestrians [16,19], it was expected that the synchronization strength in dyadic walking would be, on average, higher than that for walking in a group. Furthermore, it was expected that the instruction to synchronize steps would cause the synchronization strength to be significantly higher than in the case of lack thereof [19]. Finally, it was expected that the synchronization strength for walking front-to-back would be higher than that for walking side-by-side [16,19].

The rest of the paper is organized as follows. Section 2 introduces the developed VR platform, test subjects, experimental protocol, instrumentation and data processing. The numerical results are provided in §3. Section 4 presents the discussion of the results in the context of previous studies. Conclusions are given in §5.

## 2. Method

### 2.1. Virtual reality platform

A bespoke experimental platform was developed for the purpose of this study, relying on an immersive VR environment and a distributed motion capture system. The VR environment contained a virtual pedestrian, of which gait characteristics could be closely controlled, within a space closely resembling a real-world environment in which the experimental campaign took place. The main idea underlying the development of the experimental platform was to enable kinematic gait data to be recorded during dyadic walking with a pacer, that being either a real human or their anatomically and biomechanically representative VR avatar.

The following three main steps were implemented to create a VR platform enabling overground walking in the presence of an avatar. Firstly, walking cycles of a real person were recorded using a motion capture suit (MCS) to serve as a blueprint for the avatar’s movement. The recordings were taken with a real person walking along straights and turns at various pacing frequencies. Secondly, a realistic humanoid character to be presented in VR had to be created together with an animation controller responsible for driving the movement of the avatar. Thirdly, a state-of-the-art navigation system, using artificial intelligence, was set up and used to move the avatar within the VR environment.

#### 2.1.1. Motion capture

Eight OptiTrack Prime 13 cameras were used to record the motion of a 25-year-old male performer (height: 1.825 m, weight: 80 kg) wearing a motion capture suit equipped with 37 reflective markers placed on body landmarks. All data were recorded at the sampling rate of 120 Hz and transferred through a gigabit Ethernet network compliant with the IEEE 802.3 standard to the processing unit, where they were logged and post-processed using proprietary Motive:Body 2.1.1 software [74]. Four types of walks were performed at pacing frequencies, *f*_*p*_, ranging from 1.3 to 2.0 Hz, at 0.1 Hz increments, enforced with an audible beat of a metronome: walk in a straight line and along circular arcs with the radii of 0.635 m (25 in), 1.27 m (50 in) and 2.54 m (100 in).

The initial MCS data post-processing was done in Motive:Body software, where gaps in markers’ positions were filled using cubic or linear polynomial interpolants, depending on the trajectory of a marker. The raw data were smoothed by applying a low-pass fourth-order two-way Butterworth filter with the cut-off frequency of 6 Hz. This was done to remove any motion artefacts caused by the MCS's fabric movement and any changes in the tracking quality due to fluctuations in the lighting conditions and ambient temperature. The final stage of post-processing was performed in Autodesk MotionBuilder 2018 [75], where the MCS data were down-sampled to 30 frames s^−1^. The software enabled to adjust details, such as feet’s floor contact and fingers’ positions. Finally, to minimize the repeatability of performer’s motions and to preserve the variability of gait, multiple gait cycles recorded at the same pacing frequency and walking path during different takes were extracted. They were then stitched together in an arbitrary order to create looping sequences of performer’s motion containing an integer number of gait cycles, each slightly different than another.

#### 2.1.2. Avatar creation

A virtual representation of the performer to be incorporated within VR was created in Adobe Fuse CC 1.2 [76]. The geometry and appearance of body segments, including face expression, were chosen to match, as closely as possible, those of the performer. The character, hereafter referred to as the avatar, was then imported into (Adobe) Mixamo web service [77] where it was rigged, i.e. where its skeleton was created and tied with its geometry to facilitate the control of avatar’s motion. The avatar was then imported into Unity 2018.4.0f1 game engine [78] where a custom animation controller was built using pre-recorded performer’s walking sequences, as outlined in §2.1.1. The animation controller relied on a linear blending algorithm based on two variables: the pacing frequency and the instantaneous direction of progression. Therefore, having been prompted by a command from within a custom-written Unity3D program, the avatar could walk in a manner closely resembling the performer in terms of anatomy, biomechanics and appearance at any pacing frequency (hence speed) and in any direction, in near real time.

The auditory stepping cues were incorporated by adding a sound source to each of avatar’s feet. The sound sources were populated with a database of step sounds recorded on various walking surfaces, with 10 slightly different step sounds per surface. This allowed to play a non-repeating step sound, appropriate to the walking surface, every time the game engine detected a collision of avatar’s foot with the virtual walking surface.

#### 2.1.3. Steering system

Polarith AI 1.6 [79] was implemented to navigate the avatar around the VR scene. Polarith AI is a state-of-the-art fully programmable artificial-intelligence navigation system based on a multi-objective optimization algorithm. The workflow of the algorithm is divided into two stages. Firstly, it samples the scene to detect the position of obstacles and destination relative to the driven avatar. Secondly, it uses the inbuilt optimization algorithm to find the best local solution for the optimization problem [80]. The solution represents an optimal direction of movement which is fed directly to the avatar’s animation controller, outlined in §2.1.2. A simple ‘path follow’ action was programmed for the purpose of the tests, where the avatar was following a pre-programmed path.

### 2.2. Test subjects

The basic information about six test subjects recruited for the study is given in table 1. Before taking part in the experiment, test subjects were required to familiarize themselves with an information letter, fill in a physical activity readiness questionnaire, and sign an informed consent form. Test subjects wore casual clothing and flat sole shoes.

**Table 1.** Basic information about the test subjects of the experiment.

	test subject ID					
	S1	S2	S3	S4	S5	S6
gender	female	male	male	male	male	male
age	28	30	36	47	22	35
mass (kg)	77.0	80.1	81.6	74.4	84.9	87.8
height (cm)	172.0	191.6	173	189.5	180.5	183.5

### 2.3. Location

The Charles Wilson Sports Hall located within the main campus of the University of Leicester was chosen as an experimental location. The sports hall is 16.7 m wide and 33.5 m long and has the clear height of the ceiling at 5.6 m. The wooden floor of the hall was covered with a dark monotone carpet tiles for the purpose of the experiment, to mask the floor markings hence eliminating visual reference cues which could have affected test subjects’ behaviour.

### 2.4. Experimental protocol

Test subjects were given habituation time to familiarize themselves with the experimental environment. Each test subject was then asked to perform 16 walks, each consisting of eight laps comprising two 10 m straights and two turns along half of the circumference of a circle with 5 m in diameter. During each walk, the test subject was asked to walk together with a pacer. The pacer was either the same male subject used to animate the avatar, as described in §2.1.1, hereafter referred to as Pacer^RL^, or an animated virtual avatar, as described in §2.1.2, hereafter referred to as Pacer^VR^. Therefore, the movement of the pacer accompanying the test subject was compatible in the real-life (RL) and virtual environment (VR).

To account for any potential directional bias in pedestrian behaviour, each walk was performed in clockwise and anticlockwise directions along the looping path. Test subjects walked either next to the pacer, i.e. side-by-side on the inward of the loop, hereafter denoted SbS, or behind the pacer, i.e. front-to-back, hereafter denoted FtB. During the first eight walks, hereafter referred to as uninstructed synchronization and denoted US, the test subjects were only told to maintain their prescribed position relative to the pacer throughout the test. For the final eight walks, hereafter referred to as instructed synchronization and denoted IS, the test subjects were also instructed to walk in step with the pacer, such as to match the timing of their footsteps made with ipsilateral legs.

The pacing frequency of Pacer^RL^ was controlled by using a pair of over–ears Pioneer SE-M521 headphones connected to KORG MA-1 metronome, whereas the pacing frequency of Pacer^VR^ was directly set in the animation controller described in §2.1.2 The pacing frequency was established for each test subject individually, based on the Froude number, *FR*, equal to 0.15 and described by the following formula

$$FR=\frac{v}{gl},$$

where *v* is the walking speed (expressed in m s^−1^), *g* is the gravitational acceleration (expressed in m s^−2^) and *l* is the test subject’s leg length (expressed in m). The Froude number was used to obtain dynamically compatible gait patterns for all test subjects by compensating for differences in their anatomical (i.e. geometric) characteristics [81]. Due to practical reasons, the leg length was estimated based on the test subject’s height and gender using the relationships established by Pheasant [82] and explicitly given in Bocian *et al.* [83]

$$l=\left\{ \begin{array}{l} 0.7028\;h_{m}-0.3091\\ 0.6797\;h_{f}-0.2781 \end{array}\right.,$$

where *h*_*m*_ and *h*_*f*_ is the height for male and female test subjects (expressed in m), respectively.

Finally, the walking speed was converted to the corresponding pacing frequency, *f*_*p*_, using the relationship obtained in Soczawa-Stronczyk *et al.* [19]

$$f_{\;p}=0.66v+0.99.$$

Following this procedure resulted in test subjects’ walking with the mean speed (given with ± s.d. where applicable) of *v*^RL^ = 1.28 ± 0.07 m s^−1^ and *v*^VR^ = 1.20 ± 0.03 m s^−1^ in the RL and VR environments, respectively.

### 2.5. Instrumentation

A motion capture system consisting of 24 OptiTrack Prime 17W and 10 OptiTrack Prime 13 cameras was deployed in the sports hall. During VR tests, test subjects wore Oculus Rift CV1 head-mounted display (HMD) equipped with reflective markers to track its movement. The HMD was connected to MSI VR One 7RE backpack PC used to generate VR environment. HMD’s positional data were streamed from Motive:Body 2.1.1 software running on MCS data processing unit to the backpack PC through a wireless network compliant with IEEE 802.11n-2009 [84] standard, using the NatNet 3.0.1 [85] server broadcast protocol. This was to ensure untethered experience, i.e. for the test subjects to move freely within the capture volume of the MCS. The MCS latency, defined as the time elapsed from the cameras’ exposure to the tracking data packages fully solved by Motive software and ready for transmission over IEEE 802.11n-2009 wireless network, was measured *ex post facto*. It did not exceed 4.7 ms. The latency of the data transmission over IEEE 802.11n-2009 wireless network was estimated to be approximately 3 ms, based on the median value of a typical consumer grade, uncongested wireless network [86]. Although the latencies due to scene rendering and display in the HMD were not directly measured, a similar set-up was previously used in [43]. No discomfort due to the visual information delay was reported in *post hoc* interviews with the test subjects. If the latency had a strong debilitating effect on our results, we would have expected the mean phase difference to differ significantly between corresponding tests in RL and VR environment under the instruction to synchronize steps (IS). However, this was not the case, as discussed in §3.3.3.

**Figure 1.**
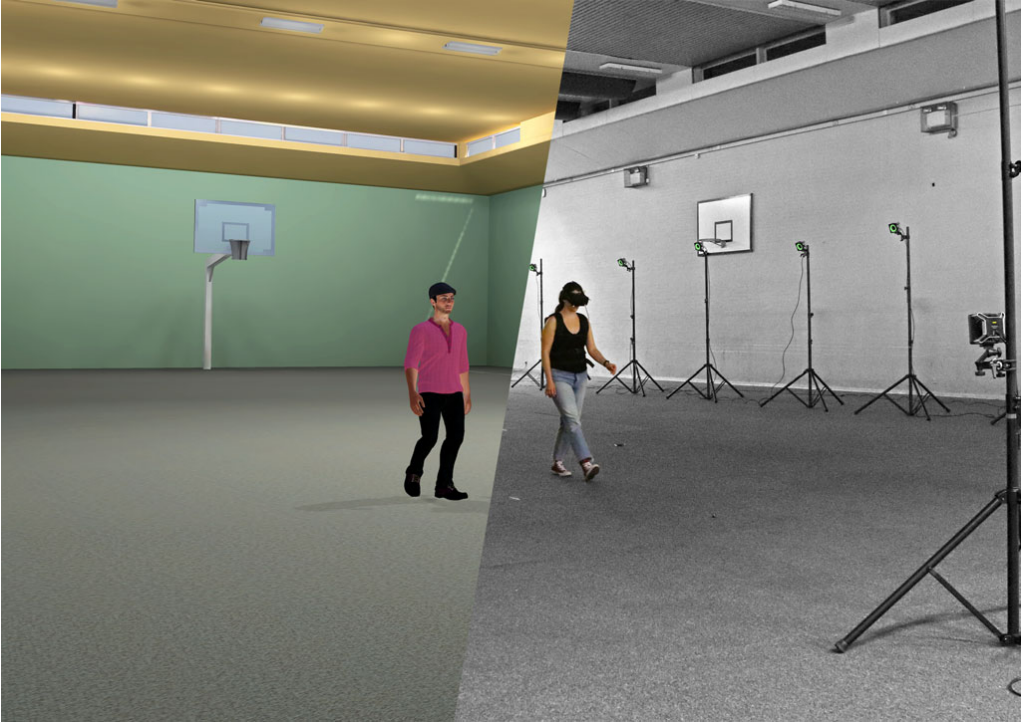
A collage with, on the right panel, test subject S1 wearing head-mounted display being tracked with a set of motion capture cameras and, on the left panel, a screenshot from virtual reality containing Pacer^VR^.

The VR environment used in the tests, shown in figure 1, was created using ARCHICAD 23 software [87]—a state-of-the-art building information modelling (BIM) tool. It consisted of a highly detailed representation of the Charles Wilson Sports Hall including three-dimensional doors, windows, lighting features and basketball infrastructure. The walls and the ceiling were covered in solid colours and the floor was rendered using a dark monotone carpet tile texture with the resolution of 288 px m^−1^. The appearance of these components was closely matching the real-world environment. To increase realism, a global (Sun) light was positioned outside the modelled room. The global light was casting inner shadows, thus providing variable brightness surface patterns facilitating optic flow and distance estimation. An exemplar VR scene used during the experimental campaign is available as a part of the dataset supporting this study (see Data accessibility).

For all the walks, test subjects were instrumented with two APDM Opal™ wireless attitude and heading reference systems (AHRS), one attached at the level of fifth lumbar vertebra and the other one on the right ankle using elastic straps. The data recorded by the AHRS were time-locked and sampled at 128 Hz. Only the data from the ankle sensor were used in subsequent analysis, as they carried information allowing unequivocal determination of the stage of a gait cycle. For this purpose, the vertical component of the acceleration vector (i.e. that aligned with gravity) was extracted by resolving the recorded three-dimensional acceleration signals from the local (i.e. sensor) to the global coordinate system using quaternion algebra.

The Pacer^RL^ was equipped with a set of AHRS of the same type and placed at the same body landmarks, and time-locked to those of the test subject thus enabling synchronization to be readily quantified based on compatible signals. However, this was impossible to realize during VR walks. In this case, the Pacer^VR^ limb’s displacement measured at the location corresponding to the placement of AHRS was recorded within the game engine at a sampling rate of approximately 50 Hz. Since that signal was not time-locked with the signals from AHRS, the following time alignment procedure was implemented. One AHRS was strapped to a rigid body tracked by the MCS and controlling the motion of an object in VR. The displacement of that virtual object was recorded by the game engine together with the displacement of the skeleton driving Pacer^VR^. Before and after each of the VR walks, the rigid body with the attached AHRS was waved slowly in sinusoidal motion to create a signal for the time alignment of data from the two systems (i.e. AHRS and VR) in post processing.

### 2.6. Data analysis

All analyses of synchronization are based on vertical velocity signals from the ankle. This is because the acceleration signals from AHRS and displacement signals from Pacer^VR^ had to be reconciled to a common physical quantity before further processing.

#### 2.6.1. Signal processing

Pacer^VR^’s signal was up-sampled from the sampling rate of approximately 50 to 128 Hz using *resample* function [88] implemented in Matlab R2019b [89], to match the sampling rate of the signals from AHRS monitors. Next, the AHRS signals (acceleration in m s^−2^) and the up-sampled signals from the game engine (displacement in m) were brought to a common kinematic variable, that being velocity (expressed in m s^−1^). To numerically integrate the AHRS’ acceleration signal, a fourth-order two-way band-pass Butterworth filter was used with the frequency band set to preserve the first three harmonics of the original signal. The signal was integrated using the cumulative trapezoidal numerical integration method [90] and then filtered using high-pass, fourth-order Butterworth filter with the cut-off frequency equal to half of the frequency of the first harmonic. The numerical differentiation of the game engine’s displacement signal was performed by calculating a one-dimensional numerical gradient of the displacement vector aligned with gravity and dividing it by the constant numerical gradient of the corresponding time. The resulting velocity vector was filtered using the fourth-order band-pass Butterworth filter with the frequency band set to preserve the first three harmonics of the signal, as in the case of numerical integration.

Having obtained signals representing the same physical quantity, it was possible to use two signals from the rigid body–AHRS couple to find the delay using *finddelay* function [88] in Matlab R2019b and align them in time.

**Figure 2.**
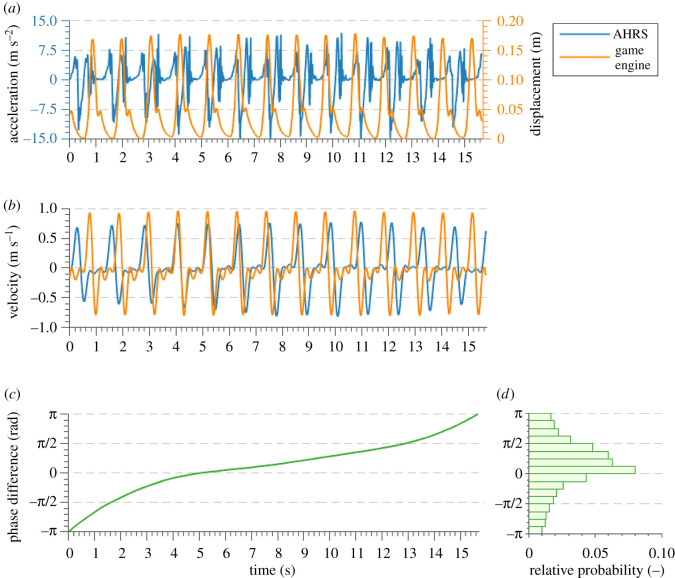
Preview of (*a*) AHRS acceleration and game engine displacement signals from the ankle, with (*b*) the results of the numerical integration and differentiation of the acceleration and displacement signals, respectively, as well as (*c*) the time series of phase difference and its (*d*) relative probability distribution. All presented signals were recorded during anticlockwise side-by-side walk with VR avatar under US experimental condition for test subject S2.

The exemplar results of the signal processing procedures described in this section are shown in figure 2*a*,*b*. Figure 2*c*,*d* is included here for the clarity of presentation, although the relevant discussion is given in §2.6.2.

Two types of signals were used in the subsequent analysis: (i) the original signals, containing the entire walking path, hereafter referred to as loops, and (ii) the truncated signals, created by removing sections of the loops containing data from the turns, hereafter referred to as straights. The locations of the individual straights in the velocity signal were identified by using the angular velocity data recorded by the AHRS attached at the test subject’s ankle.

For both types of signal, the first and the last straight sections of each walk were discarded, as they contained gait initiation and termination stages, respectively. This resulted in 15 straights per each walk type, with the exception being the virtual anticlockwise FtB walk under US experimental conditions undertaken by test subject S5 who performed seven laps around the path instead of eight.

#### 2.6.2. Quantification of synchronization

Following previous studies [8–10,12,13], an analytic signal was employed herein to derive an instantaneous phase of the captured signals. Prior to obtaining the analytic signal, the velocity signals from the ankle were band-pass filtered using the fourth-order Butterworth filter with a frequency band limited by 0.70 times the minimum stride frequency and 1.25 times the maximum stride frequency from the pair of considered walkers, as was the case in van Ulzen *et al.* [9]. The analytic signal, containing information on the instantaneous amplitude and phase angle, was obtained using Hilbert transform, commonly defined as [91]

$$v^{a}(t)=v(t)+\frac{i}{\pi }P.V.\int_{-\infty }^{+\infty }\frac{v(t^{\prime })}{t-t^{\prime }}\;\text{d}t^{\prime },$$

where *t* is the real-valued time variable, *v*(*t*) is the real-valued velocity signal, *v*^*a*^(*t*) is its analytic representation and *P*.*V*. is the Cauchy principal value of the integral. The instantaneous phase angle time series, *ϕ*(*t*), was obtained by calculating the angle between the real, R, and imaginary, I, parts of the analytic signal [92]

$$\phi (t)=\tan^{-1}\frac{I[v^{a}(t)]}{R[v^{a}(t)]}.$$

The bivariate phase difference signal, *ϕ*_*p*,*s*_(*t*), was obtained by subtracting test subject’s phase angle time series, *ϕ*_*s*_(*t*), from pacer’s phase angle time series, *ϕ*_*p*_(*t*)

$$\phi_{\;p,s}(t)=\phi_{p}(t)-\phi_{s}(t).$$

An exemplar signal representing the evolution of phase difference in time is shown in figure 2*c*, and the corresponding histogram of phase difference is shown in figure 2*d*. The rate of change of phase difference varies throughout the record, such that it is the fastest when passing through the extreme values and slows down around the state indicating no phase difference. Consequently, the phase difference distribution is skewed towards the values at and around 0 rad.

The bivariate synchronization strength index, *ρ*_*p*,*s*_, was then calculated, based on the Shannon entropy, *E*_*p*,*s*_, of the phase difference distribution [92]

$$E_{\;p,s}=-\sum_{k=1}^{N}P_{\;p,s}^{k}\ln P_{\;p,s}^{k},$$

where $P_{\;p,s}^{k}$ is the probability of the phase difference, *ϕ*_*p*,*s*_(*t*), falling into a *k*th bin of *π*/8 rad in size and *N* is the total number of bins. To allow for synchronization strength index to be comparable for different walks and test subjects, the index was normalized by the maximum attainable Shannon entropy in the case of the perfect phase synchronization

$$\rho_{\;p,s}=\frac{\ln N-E_{\;p,s}}{\ln N}.$$

The index can take values from 0 to 1, where 0 relates to a uniform distribution of phase difference, i.e. lack of synchronization, whereas 1 corresponds to Dirac-like distribution of phase difference, i.e. perfect synchronization.

## 3. Results

### 3.1. Average gait characteristics

The average stride frequency, $\;\bar{f}$, and average stride length, $\bar{l}$, were calculated for each test to evaluate the difference in gait characteristics between walking in RL and VR environment. The Welch’s *t*-test [93] at 5% significance level (*p* = 0.05) was then used to test the hypotheses that the means of *f* and *l* were equal. The data collected under US only were used in this analysis since the instruction to synchronize steps had overridden naturally occurring gait patterns, as discussed in §4.3.1 Moreover, walks in SbS and FtB were considered separately due to the difference in the distance travelled by test subjects in these two topological arrangements.

The average stride frequency, obtained using the fast Fourier transform, during walking SbS was ${\bar{\;f}}_{\mathrm{SbS}}^{\mathrm{RL}.\mathrm{US}}=0.829\pm 0.043\;\text{Hz}$ and ${\bar{\;f}}_{\mathrm{SbS}}^{\mathrm{VR}.\mathrm{US}}=0.822\pm 0.053\;\text{Hz}$ in the RL and VR environment, respectively. The corresponding values for walking FtB were ${\bar{\;f}}_{\mathrm{FtB}}^{\mathrm{RL}.\mathrm{US}}=0.832\pm 0.032\;\text{Hz}$ and ${\bar{\;f}}_{\mathrm{FtB}}^{\mathrm{VR}.\mathrm{US}}=0.852\pm 0.044\;\text{Hz}$, respectively. Hence the test subjects’ stride frequency in VR environment was on average lower by 0.77% and higher by 2.35% for walking SbS and FtB, respectively. In both cases, the difference was statistically insignificant, *p*_SbS_ = 0.748 and *p*_FtB_ = 0.226.

The average stride length for walking SbS was ${\bar{l}}_{\mathrm{SbS}}^{\mathrm{RL}.\mathrm{US}}=1.38\pm 0.10\;\text{m}$ and ${\bar{l}}_{\mathrm{SbS}}^{\mathrm{VR}.\mathrm{US}}=1.26\pm 0.09\;\text{m}$ in the RL and VR environment, respectively. The corresponding values for walking FtB were ${\bar{l}}_{\mathrm{FtB}}^{\mathrm{RL}.\mathrm{US}}=1.42\pm 0.08\;\text{m}$ and ${\bar{l}}_{\mathrm{FtB}}^{\mathrm{VR}.\mathrm{US}}=1.41\pm 0.09\;\text{m}$, respectively. Hence the test subject’s stride length in VR environment was on average lower by 8.19% and 2.00% for walking SbS and FtB, respectively. The difference was statistically significant for walking SbS, *p*_SbS_ = 0.010, and statistically insignificant for walking FtB, *p*_FtB_ = 0.402.

In a similar fashion, pacers’ average stride frequencies were extracted and compared to those of test subjects for corresponding walks. Under US experimental condition, the difference was equal to ${\bar{\;f}}_{P-S}^{\mathrm{RL}.\mathrm{US}}=0.058\pm 0.044\;\text{Hz}$ and to ${\bar{\;f}}_{P-S}^{\mathrm{VR}.\mathrm{US}}=0.052\pm 0.054$ in the RL and VR environment, respectively. Hence, on average, the pacer walked with a higher stride frequency than the test subject.

A closer match between stride frequencies was found for IS experimental condition. The average stride frequency difference was ${\bar{\;f}}_{P-S}^{\mathrm{RL}.\mathrm{IS}}=0.005\pm 0.017\;\text{Hz}$ and ${\bar{\;f}}_{P-S}^{\mathrm{VR}.\mathrm{IS}}=0.006\pm 0.017$ in RL and VR environment, respectively. Hence, on average, the pacer walked with a slightly higher stride frequency than the test subject.

### 3.2. Gait cycle variability

**Figure 3.**
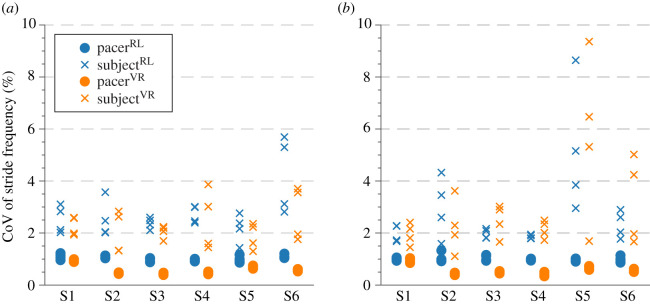
The coefficient of variation of the stride frequency of pacers and test subjects obtained (*a*) without and (*b*) with the instruction to synchronize gait.

One of the requirements of the experimental protocol was to retain similar gait variability of the pacer in all experimental conditions in order to preserve this natural gait characteristic and obtain a compatible set of results. To verify this condition, the coefficient of variation of the stride frequency, *f*_CoV_, was calculated to assess the gait variability of the pacer and all test subjects. The outcomes are shown in figure 3. Overall, the timing of the pacer’s footsteps was repetitive, which is represented by the CoV values not exceeding 1.33% during any of the walks. It is worth pointing out that, due to the way the experimental campaign was designed, the pacer’s gait variability was not directly affected by the instruction to synchronize, i.e. the change of the experimental conditions from US to IS. This is to say that the effort spent to coordinate the timing of footsteps was unidirectional, with the test subjects adjusting their steps to match those of the pacer. Moreover, the Pacer^VR^ was hard-programmed to a pre-established average pacing frequency while accounting for certain variability in the timing of footsteps (see §2.1), and by its very nature remained unaffected by test subject’s behaviour throughout the duration of the test. However, even though subtle, the gait variability difference between the human and AI-driven pacer was distinguishable. The mean CoV of the stride frequency for Pacer^RL^ is ${\bar{\;f}}_{\mathrm{CoV}.P}^{\mathrm{RL}}=1.02\pm 0.10\text{\%}$, with the minimum and maximum values of $\min\;f_{\mathrm{CoV}.P}^{\mathrm{RL}}=0.86\text{\%}$ and $\max\;f_{\mathrm{CoV}.P}^{\mathrm{RL}}=1.33\text{\%}$. The corresponding mean value for Pacer^VR^ is ${\bar{\;f}}_{\mathrm{CoV}.P}^{\mathrm{VR}}=0.59\pm 0.19\text{\%}$, with the minimum and maximum values of $\min\;f_{\mathrm{CoV}.P}^{\mathrm{VR}}=0.34\text{\%}$ and $\max\;f_{\mathrm{CoV}.P}^{\mathrm{VR}}=1.03\text{\%}$.

Overall, the gait variability of test subjects was higher than that of the pacer; however, for most of the walks the CoV of test subjects’ stride frequency did not exceed 6%. Under the US, the mean CoV of test subjects’ stride frequency was ${\bar{\;f}}_{\mathrm{CoV}.S}^{\mathrm{RL}.\mathrm{US}}=2.76\pm 0.96\text{\%}$ for walks in the RL environment and ${\bar{\;f}}_{\mathrm{CoV}.S}^{\mathrm{VR}.\mathrm{US}}=2.24\pm 0.74\text{\%}$ for walks in the VR environment. Under the IS, the mean CoV of test subjects’ stride frequency was ${\bar{\;f}}_{\mathrm{CoV}.S}^{\mathrm{RL}.\mathrm{IS}}=2.72\pm 1.56\text{\%}$ for walks in the RL environment and ${\bar{\;f}}_{\mathrm{CoV}.S}^{\mathrm{VR}.\mathrm{IS}}=2.96\pm 1.91\text{\%}$ for walks in the VR environment.

The highest gait variability under US was found for S6 in RL, at $\max\;f_{\mathrm{CoV}.S6}^{\mathrm{RL}.\mathrm{US}}=5.69\text{\%}$, and for S4 in VR, at $\max\;f_{\mathrm{CoV}.S4}^{\mathrm{VR}.\mathrm{US}}=3.87\text{\%}$. Overall, the gait variability under US did not show any systematic relationships between test subjects. The highest gait variability under IS was consistently found for S5, with $\max\;f_{\mathrm{CoV}.S5}^{\mathrm{RL}.\mathrm{IS}}=8.64\text{\%}$ and $\max\;f_{\mathrm{CoV}.S5}^{\mathrm{VR}.\mathrm{IS}}=9.36\text{\%}$ for RL and VR walks, respectively.

### 3.3. Synchronization strength

#### 3.3.1. Statistical analysis

A Wilcoxon signed-rank test [94] was performed using IBM SPSS Statistics 26 [95] to determine the effect of the type of the analysed signal (loops or straights) on the bivariate synchronization strength index. The examination of the difference scores revealed near symmetrical data distribution. The difference between the two types of the signal was not statistically significant, *z* = −1.07, *p* = 0.286. As a consequence, only the values for the truncated signals, consisting of straights, will be presented throughout this paper, to ensure a level of compatibility between the outcomes of this and previous studies carried out along straight paths.

To gain an insight into general dependencies between the bivariate synchronization strength index and the experimental conditions, the first-order multiple linear regression analysis [96] was therefore performed adopting the significance level of 5% (*p* = 0.05). The statistical model took the following factors as the explanatory variables: (i) the environment in which the test took place (real-life environment, RL, or virtual environment, VR); (ii) the presence of the instruction to synchronize (uninstructed synchronization, US, or instructed synchronization, IS); (iii) the walking direction (clockwise or anticlockwise); and (iv) the relative position of the walkers (side-by-side, SbS, or front-to-back, FtB). The results showed that the presence of the instruction to synchronize had the strongest influence on the synchronization strength index, *t* = 17.92, *p* < 0.0005. This was followed by a much weaker, yet statistically significant influence of the environment, *t* = −2.04, *p* = 0.045. The remaining explanatory variables were not statistically significant predictors of the synchronization strength index. Therefore, from here onwards, the data will be presented separately for each of the environmental and experimental conditions and the walkers’ collocation. No distinction will be made between clockwise and anticlockwise walking direction.

**Figure 4.**
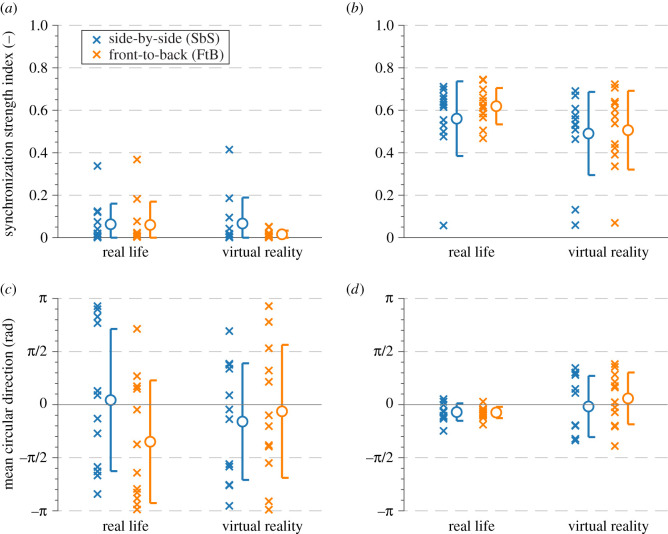
The results in terms of (*a*,*b*) synchronization strength indices and (*c*,*d*) mean circular direction values obtained (*a*,*c*) without and (*b*,*d*) with the instruction to synchronize gait. The values are presented with their respective means (denoted by circles) and ± s.d. intervals (denoted by whiskers).

The synchronization strength indices are presented in figure 4*a*,*b* and accompanied by the mean circular direction indicating the directionality of the phase difference distribution in figure 4*c*,*d*. The mean circular direction, $\bar{\mu }$, was calculated by transforming all phase difference values into a two-dimensional vector ***μ*** = (cos *α*, sin *β*) and averaging over the number of data points [97]. According to the adopted convention, negative and positive values indicate the test subject is lagging and leading the pacer, respectively.

#### 3.3.2. Uninstructed synchronization

The mean values of the synchronization strength index obtained for US in the two visual environments are rather low. In the RL environment, these values are ${\bar{\rho }}_{\mathrm{SbS}}^{\mathrm{RL}.\mathrm{US}}=0.063\pm 0.097$ and ${\bar{\rho }}_{\mathrm{FtB}}^{\mathrm{RL}.\mathrm{US}}=0.060\pm 0.110$ for walking SbS and FtB, respectively. In the VR environment, the corresponding values are ${\bar{\rho }}_{\mathrm{SbS}}^{\mathrm{VR}.\mathrm{US}}=0.067\pm 0.122$ and ${\bar{\rho }}_{\mathrm{FtB}}^{\mathrm{VR}.\mathrm{US}}=0.016\pm 0.018$. Only three synchronization strength indices reach above 0.21, previously suggested to signify the synchronization threshold for walking in pairs [12].

The directionality of synchronization, quantified by the mean circular direction, is ${\bar{\mu }}_{\mathrm{SbS}}^{\mathrm{RL}.\mathrm{US}}=0.04\pi \pm 0.67\pi \;\text{rad}$ and ${\bar{\mu }}_{\mathrm{FtB}}^{\mathrm{RL}.\mathrm{US}}=-0.35\pi \pm 0.58\pi \;\text{rad}$ for walking SbS and FtB in RL, respectively. In the VR, the corresponding values are ${\bar{\mu }}_{\mathrm{SbS}}^{\mathrm{VR}.\mathrm{US}}=-0.16\pi \pm 0.55\pi \;\text{rad}$ and ${\bar{\mu }}_{\mathrm{FtB}}^{\mathrm{VR}.\mathrm{US}}=-0.06\pi \pm 0.63\pi \;\text{rad}$. Considering the results for both visual environments, the negative and positive values of the mean circular direction were recorded in 58.3% and 41.7% of cases, respectively.

#### 3.3.3. Instructed synchronization

The mean values of the synchronization strength index for all walks obtained for IS exceed 0.21, previously suggested to signify the synchronization threshold for walking in pairs [12]. In the real environment, these values are ${\bar{\rho }}_{\mathrm{SbS}}^{\mathrm{RL}.\mathrm{IS}}=0.560\pm 0.176$ and ${\bar{\rho }}_{\mathrm{FtB}}^{\mathrm{RL}.\mathrm{IS}}=0.619\pm 0.086$ for walking SbS and FtB, respectively. In the virtual environment, the corresponding values are ${\bar{\rho }}_{\mathrm{SbS}}^{\mathrm{VR}.\mathrm{IS}}=0.490\pm 0.196$ and ${\bar{\rho }}_{\mathrm{FtB}}^{\mathrm{VR}.\mathrm{IS}}=0.506\pm 0.186$. The synchronization strength index values obtained for walking in RL are, on average, higher by 15.6% than their VR counterparts. Considering the results for both visual environments, the highest mean values of the synchronization strength index were obtained for walking FtB.

In the real environment, the mean values of the mean circular direction are ${\bar{\mu }}_{\mathrm{SbS}}^{\mathrm{RL}.\mathrm{IS}}=-0.07\pi \pm 0.08\pi$ rad and ${\bar{\mu }}_{\mathrm{FtB}}^{\mathrm{RL}.\mathrm{IS}}=-0.07\pi \pm 0.05\pi$ rad for walking SbS and FtB, respectively, with 91.7% of data points taking negative values. In the virtual environment, the corresponding values are ${\bar{\mu }}_{\mathrm{SbS}}^{\mathrm{VR}.\mathrm{IS}}=-0.02\pi \pm 0.29\pi \;\text{rad}$ and ${\bar{\mu }}_{\mathrm{FtB}}^{\mathrm{VR}.\mathrm{IS}}=0.06\pi \pm 0.24\pi$ rad, with only 45.8% of data points taking negative values.

## 4. Discussion

### 4.1. Average gait characteristics

The average stride frequency and stride length of test subjects for walking SbS and FtB under US was similar between the corresponding tests conducted in RL and VR environment. This is apart from the average stride length in walking SbS for which there was a statistically significant difference. However, this occurrence cannot be considered as evidence supporting the notion of gait parameters being adversely affected by the VR immersion. This is because the average duration of tests in RL and VR environment was in this case different, at 206 and 230 s, respectively. This is not the case for walking FtB, for which the average duration of tests in RL and VR environment was similar, at 231 and 228 s, respectively. In both cases (SbS and FtB walking), the difference must be predominantly attributed to the intra-subject variability of the pacer. This is because the requirement of accompanied walking imposed by the experimental protocol determined the walking speed of the test subjects, hence their stride frequency and stride length, during each test. This is to say that the gait characteristics exhibited by the pacer during recording of the walking gait data for use in the avatar animation (i.e. Pacer^VR^), as described in §2.1.1, and those during the tests in RL (i.e. Pacer^RL^) differed. Therefore, the comparison of average gait parameters for walking SbS is not informative as to the influence of VR on gait characteristics.

Overall, it is concluded that the effect of reduction in gait parameters pertaining to the spatial and temporal reference frames, associated with VR immersion [36,62–68], is not evident in the obtained results. However, it needs to be borne in mind that the current study was not designed to address this particular problem.

### 4.2. Gait variability

The values of gait variability obtained for the pacer are slightly lower than the average gait variability for overground walking at normal speeds [98]. This is deemed acceptable since the protocol requirement of the gait of the pacer to exhibit some variability had to be reconciled with the requirement of having to produce a gait rhythm at the predefined (mean) frequency. Another acceptable circumstance is the difference between mean CoV of stride frequency of the real-life and virtual pacer at 0.42%.

The analysis of the gait variability of test subjects did not highlight any noteworthy dissimilarities between the two experimental conditions, except S5 exhibiting more variable gait in the VR environment.

### 4.3. Synchronization strength

The effect of the instruction to synchronize steps on the synchronization strength index obtained for walking in the VR environment is comparable to that obtained in the RL environment. This is the case for both considered topological arrangements (i.e. SbS and FtB). However, quantitative and qualitative differences were found between gait coordination patterns from corresponding tests in the RL and VR environments.

#### 4.3.1. The effect of the instruction to synchronize steps

The mean synchronization strength indices obtained in the RL and VR environments are comparable under US for walking SbS. For walking FtB, the index obtained in the VR environment is much lower than that obtained in the RL environment. The magnitude of synchronization strength index under US is generally low, and there is a considerable spread of the mean circular direction data, suggesting the behaviour of pedestrians is highly random and any persistent gait coordination patterns are seldom present. This is unlike the results for walking under IS.

The mean synchronization strength indices under IS are much higher than those obtained under US and so is the variability of the results. This is due to the conscious effort spent on gait coordination, as dictated by the experimental protocol, and considerable intra- and inter-subject variability in this respect. The synchronization strength index for walking FtB is consistently higher than that for walking SbS for tests conducted in the same visual conditions. This agrees with the results of previous investigations probing the influence of pedestrians’ topological arrangement on the synchronization strength for walking in the RL environment [16,19].

#### 4.3.2. Comparison with previous studies on walking in dyads

The vast majority of walks performed under the US was characterized by low synchronization strength index, much below the theorized synchronization threshold of 0.21 [12]. This is in line with findings from van Ulzen *et al.* [9,10]. However, the current results stand in opposition to those from some other studies reporting that the gait synchronization in walking dyads is rather widespread, with synchronization signatures present in 25–70% of performed walks, as shown in table 2. This is believed to be caused by insufficiently long walking paths adopted in those studies, ranging from 15 to 70 m. In comparison, each test subject covered the distance of approximately 286 m along the entire walking path (approx. 150 m of straight sections) during a single walk in this study, and 593 m in the study reported by van Ulzen *et al.* [9], albeit conducted for walking on a treadmill rather than overground. The problem with the quantification of synchronization strength based on the phase difference distribution, using signals of short duration, is illustrated here based on a theoretical argument and the data collected in this study.

**Table 2.** The probability of occurrence of synchronization in dyadic walking after selected authors.

author	no. pairs	mode	distance/ duration	quantification method	results	comments
Chambers *et al.* [4]	441	side by side, overground	up to 30 s	estimation of ankles’ vertical displacement based on Youtube videos	35–70%—auditory and visual cues	
Harrison & Richardson [14]	6	front to back, overground	35 m	Knees’ flexion and extension using electrogoniometers	40%—auditory and visual cues	
Nessler & Gilliland [5]	20	side by side, treadmill	69.6 ± 3.6 m*	Ankles’ trajectories on the sagittal plane using MCS	33.4±30.1%—auditory and visual cues	
Nessler *et al.* [6]	24	tide by side, treadmill	60 s	right lateral malleolus’ trajectory using MCS	50%—auditory and visual cues	
van Ulzen *et al.* [9]	11	side by side, treadmill	593 m*	lower legs’ movements using movement registration system	transient episodes in up to 36% of pairs—auditory and visual cues	variable walking speed
Zivotofsky & Hausdorff [11]	14	side by side, overground	15 m	visual assessment of lower extremities’ motion by two physical therapists	29%—auditory only; 7%—visual only	obstructed cues
Zivotofsky *et al.* [12]	14	side by side, overground	70 m	vertical accelerations of lower backs using accelerometers attach at L5	36%—auditory only; 14%—visual only	obstructed cues
Zivotofsky *et al.* [13]	16	side by side, overground	70 m	vertical accelerations of lower backs using accelerometers attach at L5	25%—auditory and visual cues	

*Calculated based on the test duration and walking speed values given by the original authors.

The evidence suggests [9,10,18] that the coordination of gait between a pair of walkers is most often not a persistent occurrence, which can be maintained throughout an extended period. Instead, gait synchronization emerges spontaneously, and it dies out after a few gait cycles. This transient nature of the phenomenon, together with a relatively narrow range of pedestrian stride frequencies adopted in walking gait, can be the root cause of a systematic error in the quantification of synchronization strength index.

If the analysed gait signals are too short, the most likely outcome is the apparent high values of synchronization strength index. This is because the evolution of phase difference in time for two pedestrians walking with similar, but not the same, stride frequencies is relatively slow. For example, imagine two pedestrians walking at the same speed next to each other, hence their walk having the dyadic property, but adopting different, time-invariant stride frequencies: 0.96 and 0.98 Hz. Therefore, the expected synchronization strength index is zero. Since the synchronization strength index relies on a histogram of phase difference, this is only true if the distribution is uniform. Assuming sufficiently high sampling rate of the recorded (digital) signals, this condition is met if the duration of the analysed record is 50 s or its integer multiples. The error in synchronization strength estimates due to the deviation from this condition will generally diminish with the signal length. However, the effect of the insufficient test duration will be particularly debilitating in the case of tests lasting less than 50 s. Assuming the pedestrians’ walking speed is close to that typically observed for normal walking, e.g. 1.5 m s^−1^, this corresponds to the distance of 75 m.

Now, consider the synchronization strength indices calculated separately for each 10 m long straight section of the path used in this study, corresponding to approximately seven full gait cycles. The synchronization strength index under US is then 0.279 ± 0.251 and 0.289 ± 0.215 for walking in the RL and VR environments, respectively, which is above the proposed synchronization threshold [12]. This would lead to a false conclusion that the gait synchronization was ubiquitously present during US tests. On the other hand, under the IS, where the gait coordination was genuinely present, the analysis of individual straights would yield the mean synchronization strength indices higher by 20% and 30% for walking in the RL and VR environments, respectively, from those obtained by aggregating phase difference data from all straights prior to further processing.

It is concluded, based on these arguments, that the coordination of gait in overground walking in dyads with a real or virtual pacer, in the absence of sensory cues promoting synchronization other than visual and auditory, is relatively weak under US.

#### 4.3.3. Dyad versus group walking

The synchronization strength indices obtained for dyad walking under IS are approximately 50% higher than those previously reported from a group of walkers [19]. This can be mainly attributed to the attention tuning. The dominant focus of attention during dyad walking in an experimental environment characterized by low-complexity, which is the case for most laboratory-based investigations, is the fellow pedestrian. The visual and auditory cues provided by the fellow pedestrian constitute relatively strong stimulus for gait coordination. By contrast, concurrent sensory stimuli from multiple pedestrians are available during walking in a group, with the most prominent gait coordination cues generated by the pedestrians in the immediate vicinity of the observer [19]. The abundance of sensory information, in this case, tends to break any persistent gait coordination patterns.

#### 4.3.4. Qualitative differences between IS tests in RL and VR

Although the difference in the magnitude of mean synchronization strength indices for corresponding tests in RL and VR environments under IS is small (figure 4), it is accompanied by a distinct qualitative change in the pedestrian behaviour. On the one hand, approximately 92% of the walks performed in the RL environment are characterized by negative values of the mean circular direction. According to the sign convention adopted in this study, this means that the test subjects were most often lagging behind the pacer. On the other hand, approximately 54% of the corresponding walks in the VR environment are characterized by positive values of the mean circular direction, indicating the test subjects were, slightly more often, leading the pacer.

Given the unidirectional nature of the interaction, i.e. the Pacer^VR^ not reacting to any actions in its surroundings, this could be a consequence of the test subjects’ anticipatory behaviour. It was previously speculated that such behaviour could emerge as a result of the collision avoidance mechanism present in walking FtB [99]. In order to enable more efficient gait corrections, the follower would try to arrive at the double stance phase of gait faster than the pedestrian positioned in front. This, however, does not explain the presence of anticipatory gait behaviour for walking SbS.

It was observed during some of the SbS tests in the VR, although not measured directly, that the test subject either tried to fall back slightly behind the pacer, so that the pacer was within their sight, or turned their head towards the pacer. This intermittent behaviour might have been the result of the limited horizontal field of view (FoV) provided by the HMD, confined to around 90 degrees [100]. In comparison, the unobstructed FoV of an average human spans approximately 200 degrees in the horizontal direction [101], which is significantly more than that of the HMD. It was previously shown that limiting horizontal FoV can impact one’s perception of space [102], which might have led to more conscious stepping behaviour.

Another possible explanation of the anticipatory behaviour during VR walks is associated with the repetitiveness of Pacer^VR^’s motions which allowed for greater predictability of its gait cycle. The effect could have been magnified by more secluded conditions offered by the virtual environment. Although the VR environment was designed to be a near-identical copy of the real space where the experimental campaign took place, VR isolated test subjects from peripheral stimuli (i.e. the research team supervising the experimental procedure) present in the RL environment. The lack of peripheral stimuli decreased test subjects’ cognitive load and allowed for greater focus on Pacer^VR^’s movements. However, this was not reflected by an increase of the synchronization strength index in VR environment, as the index took lower values than in RL environment.

#### 4.3.5. Limitations and future work

The main limitations of the VR technology used in this study are associated with the relatively narrow FoV provided by the current generation of consumer-grade HMD, test subjects lacking the self-embodiment, i.e. virtual body presence, and unidirectional gait adaptation. Whether or not the lack of body presence can significantly affect gait in virtual environments remains unclear [35,103]. However, it is believed to contribute to the discrepancy in the performance of tasks requiring body coordination, such as those considered herein. The interaction with the pacer was unidirectional in the current study, whereas in the real world the interaction for walking in dyads would probably exhibit signs of bidirectionality.

The limited extent of visual field can be addressed by using a new generation of HMDs with a high refresh rate and wide FoV in vertical and horizontal directions. The self-embodiment can be provided by employing a greater number of motion capture cameras to minimize the influence of tracking markers’ occlusions on the tracking quality, hence body reconstruction, or by fusing data from other instrumentation systems. Another layer of complexity can be added to the AI-driven virtual avatar by implementing bidirectional gait coordination with the neighbouring real walker. This would, however, require a deeper understanding of the bidirectional interactions while walking in dyads. Finally, the simulations can be extended to include a crowd of intelligent virtual walkers interacting with the VR-immersed real walker. Work is currently underway to realize this ambition.

## 5. Conclusion

The spontaneous gait coordination between a pair of pedestrians, in the absence of sensory cues promoting synchronization other than visual and auditory, is generally weak. The mean synchronization strength index derived from Shannon entropy reaches up to 0.07. This is consistent regardless of the type of visual environment and pedestrians’ topological arrangement applied during the tests. Any gait synchronization patterns, if present, are in this case of transient nature and the mode of synchronization shows high variability, consistent between all walking conditions.

The instruction to synchronize gait significantly increases the synchronization strength index regardless of the type of visual environment and pedestrians’ topological arrangement applied during the tests. However, quantitative and qualitative differences are found between results for walking in the real-life and virtual environment. The mean synchronization strength index is generally higher for walking with a real rather than virtual pacer, reaching 0.56 and 0.62 for walking side-by-side and front-to-back, respectively. The corresponding values for walking in the virtual environment are 0.49 and 0.51. Walking in front-to-back arrangement consistently yields higher synchronization strength index than walking side-by-side for both visual environments. Walking in the virtual environment is characterized by higher variability of the synchronization strength index than walking in the real-life environment. The test subjects lag the pacer in close to 92% of the cases while walking in the real-life environment, but they lead the pacer in just over 54% of the cases while walking in the virtual environment. Although this may seem like a significant qualitative difference, the quantitative difference in mean circular direction is relatively small. The average values in the real-life environment are −0.07π rad both for walking side-by-side and front-to-back. The corresponding values in the virtual environment are −0.02π and 0.06π rad. The main difference lies in the variability of the mean circular direction, which is 3.6 and 4.8 times higher for walking side-by-side and front-to-back, respectively, in the virtual reality when quantified in terms of the standard deviation. The observed effect is probably due to the lack of self-embodiment in the virtual environment and limited field of view of the head-mounted display.

Overall, the results presented herein support the notion of the VR technology showing high promise for human locomotion studies, in the context of interpersonal gait coordination. Work is currently underway to extend the investigations on locomotion in virtual reality from walking in dyads to groups and crowds of pedestrians represented as intelligent avatars.

## Supplementary Material

Reviewer comments

## Data accessibility

The dataset supporting this study is available online at the Dryad Digital Repository: https://doi.org/10.5061/dryad.vx0k6djnr [104].

## References

[1] Dallard P, Fitzpatrick AJ, Flint A, Le Bourva S, Low A, Ridsdill Smith RM, Willford M 2001 The London millennium footbridge. Struct. Eng. 79, 17–33.

[2] Dallard P, Fitzpatrick T, Flint A, Low A, Smith RR, Willford M, Roche M 2001 London millennium bridge: pedestrian-induced lateral vibration. J. Bridge Eng. 6, 412–417. (10.1061/(ASCE)1084-0702(2001)6:6(412))

[3] Venuti F, Bruno L 2009 Crowd-structure interaction in lively footbridges under synchronous lateral excitation: a literature review. Phys. Life Rev. 6, 176–206. (10.1016/j.plrev.2009.07.001) 20416851

[4] Chambers C, Kong G, Wei K, Kording K 2019 Pose estimates from online videos show that side-by-side walkers synchronize movement under naturalistic conditions. PLoS ONE 14, e0217861 (10.1371/journal.pone.0217861) 31170214 PMC6553729

[5] Nessler JA, Gilliland SJ 2009 Interpersonal synchronization during side by side treadmill walking is influenced by leg length differential and altered sensory feedback. Hum. Mov. Sci. 28, 772–785. (10.1016/j.humov.2009.04.007) 19796834

[6] Nessler JA, Kephart G, Cowell J, De Leone CJ 2011 Varying treadmill speed and inclination affects spontaneous synchronization when two individuals walk side by side. J. Appl. Biomech. 27, 322–329. (10.1123/jab.27.4.322) 21896952

[7] Noy D, Mouta S, Lamas J, Basso D, Silva C, Santos JA 2017 Audiovisual integration increases the intentional step synchronization of side-by-side walkers. Hum. Mov. Sci. 56, 71–87. (10.1016/j.humov.2017.10.007) 29107820

[8] Sylos-Labini F, D’Avella A, Lacquaniti F, Ivanenko Y 2018 Human-human interaction forces and interlimb coordination during side-by-side walking with hand contact. Front. Physiol. 9, 179 (10.3389/fphys.2018.00179) 29563883 PMC5850283

[9] van Ulzen NR, Lamoth CJ, Daffertshofer A, Semin GR, Beek PJ 2008 Characteristics of instructed and uninstructed interpersonal coordination while walking side-by-side. Neurosci. Lett. 432, 88–93. (10.1016/j.neulet.2007.11.070) 18242846

[10] van Ulzen NR, Lamoth CJ, Daffertshofer A, Semin GR, Beek PJ 2010 Stability and variability of acoustically specified coordination patterns while walking side-by-side on a treadmill: does the seagull effect hold?. Neurosci. Lett. 474, 79–83. (10.1016/j.neulet.2010.03.008) 20226230

[11] Zivotofsky AZ, Hausdorff JM 2007 The sensory feedback mechanisms enabling couples to walk synchronously: an initial investigation. J. Neuroeng. Rehabil. 4, 5 (10.1186/1743-0003-4-28) 17686150 PMC1973071

[12] Zivotofsky AZ, Gruendlinger L, Hausdorff JM 2012 Modality-specific communication enabling gait synchronization during over-ground side-by-side walking. Hum. Mov. Sci. 31, 1268–1285. (10.1016/j.humov.2012.01.003) 22727358

[13] Zivotofsky AZ, Bernad-Elazari H, Grossman P, Hausdorff JM 2018 The effects of dual tasking on gait synchronization during over-ground side-by-side walking. Hum. Mov. Sci. 59, 20–29. (10.1016/j.humov.2018.03.009) 29579621

[14] Harrison SJ, Richardson MJ 2009 Horsing around: spontaneous four-legged coordination. J. Mot. Behav. 41, 519–524. (10.3200/35-08-014) 19567365

[15] Marmelat V, Delignières D, Torre K, Beek PJ, Daffertshofer A 2014 ’Human paced’ walking: followers adopt stride time dynamics of leaders. Neurosci. Lett. 564, 67–71. (10.1016/j.neulet.2014.02.010) 24548624

[16] Bocian M, Brownjohn JMW, Racic V, Hester D, Quattrone A, Gilbert L, Beasley R 2018 Time-dependent spectral analysis of interactions within groups of walking pedestrians and vertical structural motion using wavelets. Mech. Syst. Signal Process. 105, 502–523. (10.1016/j.ymssp.2017.12.020)

[17] Pimentel RL, Araújo MC, Brito HMBF, de Brito JLV 2013 Synchronization among pedestrians in footbridges due to crowd density. J. Bridge Eng. 18, 400–408. (10.1061/(ASCE)BE.1943-5592.0000347)

[18] Ricciardelli F, Pansera A 2010 An experimental investigation into the interaction among walkers in groups and crowds. In *Proc. of the 10th Int. Conf. on Recent Advances in Structural Dynamics* Southampton, UK: University of Southampton.

[19] Soczawa-Stronczyk AA, Bocian M, Wdowicka H, Malin J 2019 Topological assessment of gait synchronisation in overground walking groups. Hum. Mov. Sci. 66, 541–553. (10.1016/j.humov.2019.06.007) 31220693

[20] Alton F, Baldey L, Caplan S, Morrissey MC 1998 A kinematic comparison of overground and treadmill walking. Clin. Biomech. 13, 434–440. (10.1016/S0268-0033(98)00012-6) 11415818

[21] Stolze H, Kuhtz-Buschbeck JP, Mondwurf C, Boczek-Funcke A, Jöhnk K, Deuschl G, Illert M 1997 Gait analysis during treadmill and overground locomotion in children and adults. Electroencephalogr. Clin. Neurophysiol. 105, 490–497. (10.1016/S0924-980X(97)00055-6) 9448652

[22] Mallot HA, Gillner S, van Veen HAHC, Bülthoff HH 1998 Behavioral experiments in spatial cognition using virtual reality. In *Spatial cognition: an interdisciplinary approach to representing and processing spatial knowledge* (eds C Freksa, C Habel, KF Wender), pp. 447–467. Berlin, Heidelberg: Springer (10.1007/3-540-69342-4_21)

[23] Mohler BJ, Creem-Regehr SH, Thompson WB 2006 The influence of feedback on egocentric distance judgments in real and virtual environments. In *Proc. – APGV 2006: Symp. on Applied Perception in Graphics and Visualization*, pp. 93–100. New York, NY: Association for Computing Machinery (10.1145/1140491.1140493)

[24] Ruddle RA, Péruch P 2004 Effects of proprioceptive feedback and environmental characteristics on spatial learning in virtual environments. Int. J. Human Comput. Stud. 60, 299–326. (10.1016/j.ijhcs.2003.10.001)

[25] Fung J, Richards CL, Malouin F, McFadyen BJ, Lamontagne A 2006 A treadmill and motion coupled virtual reality system for gait training post-stroke. Cyberpsychol. Behav. 9, 157–162. (10.1089/cpb.2006.9.157) 16640470

[26] Jaffe DL, Brown DA, Pierson-Carey CD, Buckley EL, Lew HL 2004 Stepping over obstacles to improve walking in individuals with poststroke hemiplegia. J. Rehabil. Res. Dev. 41, 283–292. (10.1682/JRRD.2004.03.0283) 15543446

[27] Kim A, Schweighofer N, Finley JM 2019 Locomotor skill acquisition in virtual reality shows sustained transfer to the real world. J. Neuroeng. Rehabil. 16, 113–123. (10.1186/s12984-019-0584-y) 31521167 PMC6744642

[28] Mirelman A, Maidan I, Herman T, Deutsch JE, Giladi N, Hausdorff JM 2011 Virtual reality for gait training: can it induce motor learning to enhance complex walking and reduce fall risk in patients with Parkinson’s disease? J. Gerontol. Ser. A Biol. Sci. Med. Sci. 66A, 234–240. (10.1093/gerona/glq201) 21106702

[29] Mirelman A *et al.* 2016 Addition of a non-immersive virtual reality component to treadmill training to reduce fall risk in older adults (V-TIME): a randomised controlled trial. The Lancet 388, 1170–1182. (10.1016/S0140-6736(16)31325-3) 27524393

[30] Parijat P, Lockhart TE, Liu J 2015 Effects of perturbation-based slip training using a virtual reality environment on slip-induced falls. Ann. Biomed. Eng. 43, 958–967. (10.1007/s10439-014-1128-z) 25245221 PMC4384510

[31] Rizzo A, Kim GJ 2005 A SWOT analysis of the field of virtual reality rehabilitation and therapy. Presence: Teleoperators Virtual Environ. 14, 119–146. (10.1162/1054746053967094)

[32] Shema SR *et al.* 2014 Clinical experience using a 5-week treadmill training program with virtual reality to enhance gait in an ambulatory physical therapy service. Phys. Ther. 94, 1319–1326. (10.2522/ptj.20130305) 24786944

[33] Yang YR, Tsai MP, Chuang TY, Sung WH, Wang RY 2008 Virtual reality-based training improves community ambulation in individuals with stroke: a randomized controlled trial. Gait Posture 28, 201–206. (10.1016/j.gaitpost.2007.11.007) 18358724

[34] Agethen P, Sekar VS, Gaisbauer F, Pfeiffer T, Otto M, Rukzio E 2018 Behavior analysis of human locomotion in the realworld and virtual reality for the manufacturing industry. ACM Trans. Appl. Percept. 15, 1–19. (10.1145/3230648)

[35] Canessa A, Casu P, Solari F, Chessa M 2019 Comparing real walking in immersive virtual reality and in physical world using gait analysis. In *VISIGRAPP 2019 – Proc. of the 14th Int. Joint Conf. on Computer Vision, Imaging and Computer Graphics Theory and Applications*, vol. 2, pp. 121–128. Setúbal, Portugal: SCITEPRESS – Science and Technology Publications (10.5220/0007380901210128)

[36] Janeh O, Langbehn E, Steinicke F, Bruder G, Gulberti A, Poetter-Nerger M 2017 Walking in virtual reality: effects of manipulated visual self-motion on walking biomechanics. ACM Trans. Appl. Percept. 14, 1–15. (10.1145/3022731)

[37] Multon F, Olivier AH 2013 Biomechanics of walking in real world: naturalness we wish to reach in virtual reality. In *Human walking in virtual environments: perception, technology, and applications* (eds F Steinicke, Y Visell, J Campos, A Lécuyer), pp. 55–77. New York, NY: Springer. (10.1007/978-1-4419-8432-6_3)

[38] Steinicke F, Visell Y, Campos JL, Lécuyer A 2013 *Human walking in virtual environments: perception, technology, and applications*. New York, NY: Springer. (10.1007/978-1-4419-8432-6)

[39] Suma EA, Bruder G, Steinicke F, Krum DM, Bolas M 2012 A taxonomy for deploying redirection techniques in immersive virtual environments. In *Proc. – IEEE Virtual Reality*, pp. 43–46. IEEE (10.1109/VR.2012.6180877)

[40] Nabiyouni M, Saktheeswaran A, Bowman DA, Karanth A 2015 Comparing the performance of natural, semi-natural, and non-natural locomotion techniques in virtual reality. In *2015 IEEE Symp. on 3D User Interfaces, 3DUI 2015 – Proc.*, pp. 3–10. IEEE (10.1109/3DUI.2015.7131717)

[41] Ruddle RA, Lessels S 2009 The benefits of using a walking interface to navigate virtual environments. ACM Trans. Comput.-Human Interact. 16, 1–18. (10.1145/1502800.1502805)

[42] Ruddle RA, Volkova E, Bülthoff HH 2013 Learning to walk in virtual reality. ACM Trans. Appl. Percept. 10, 1–17. (10.1145/2465780.2465785)

[43] Berton F, Olivier AH, Bruneau J, Hoyet L, Pettre J 2019 Studying gaze behaviour during collision avoidance with a virtual walker: influence of the virtual reality setup. In *26th IEEE Conf. on Virtual Reality and 3D User Interfaces, VR 2019 – Proc.*, pp. 717–725. IEEE. (10.1109/VR.2019.8798204)

[44] Olivier AH, Bruneau J, Kulpa R, Pettre J 2018 Walking with virtual people: evaluation of locomotion interfaces in dynamic environments. IEEE Trans. Vis. Comput. Graph 24, 2251–2263. (10.1109/TVCG.2017.2714665) 28613177

[45] Olivier AH, Bruneau J, Cirio G, Pettré J 2014 A virtual reality platform to study crowd behaviors. *Transportation Research Procedia* **2**, 114–122 (10.1016/j.trpro.2014.09.015)

[46] Moussaïd M, Schinazi VR, Kapadia M, Thrash T 2018 Virtual sensing and virtual reality: how new technologies can boost research on crowd dynamics. Front. Rob. AI 5, 82 (10.3389/frobt.2018.00082) PMC7806084 33500961

[47] Boletsis C, Cedergren JE 2019 VR locomotion in the new era of virtual reality: an empirical comparison of prevalent techniques. Adv. Human-Comput. Interact. 2019, 1–15. (10.1155/2019/7420781)

[48] Templeman JN, Denbrook PS, Sibert LE 1999 Virtual locomotion: walking in place through virtual environments. Presence: Teleoperators Virtual Environ. 8, 598–617. (10.1162/105474699566512)

[49] Usoh M, Arthur K, Whitton MC, Bastos R, Steed A, Slater M, Brooks FP 1999 Walking > walking-in-place > flying, in virtual environments. In *Proc. of the 26th Annual Conf. on Computer Graphics and Interactive Techniques, SIGGRAPH 1999*, pp. 359–364. New York, NY: Association for Computing Machinery, Inc (10.1145/311535.311589)

[50] Bocian M, Macdonald JH, Burn JF, Redmill D 2015 Experimental identification of the behaviour of and lateral forces from freely-walking pedestrians on laterally oscillating structures in a virtual reality environment. Eng. Struct. 105, 62–76. (10.1016/j.engstruct.2015.09.043) 28018012 PMC5180595

[51] Bouguila L, Ishii M, Sato M 2002 Realizing a new step-in-place locomotion interface for virtual environment with large display system. In *Proc. of the Workshop on Virtual Environments 2002**EGVE ’02, Barcelona, Spain, May*, pp. 197–207. Goslar, Germany: Eurographics Association (10.2312/EGVE/EGVE02/197-207)

[52] McAndrew PM, Dingwell JB, Wilken JM 2010 Walking variability during continuous pseudo-random oscillations of the support surface and visual field. J. Biomech. 43, 1470–1475. (10.1016/j.jbiomech.2010.02.003) 20346453 PMC2866814

[53] McAndrew PM, Wilken JM, Dingwell JB 2011 Dynamic stability of human walking in visually and mechanically destabilizing environments. J. Biomech. 44, 644–649. (10.1016/j.jbiomech.2010.11.007) 21094944 PMC3042508

[54] McAndrew Young PM, Wilken JM, Dingwell JB 2012 Dynamic margins of stability during human walking in destabilizing environments. J. Biomech. 45, 1053–1059. (10.1016/j.jbiomech.2011.12.027) 22326059 PMC3321251

[55] Schwaiger M, Thümmel T, Ulbrich H 2007 Cyberwalk: implementation of a ball bearing platform for humans. In *Human-computer interaction: interaction platforms and techniques* (ed. JA Jacko), pp. 926–935. Berlin, Heidelberg: Springer (10.1007/978-3-540-73107-8_102)

[56] Souman JL, Giordano PR, Schwaiger M, Frissen I, Thümmel T, Ulbrich H, Luca AD, Bülthoff HH, Ernst MO 2008 CyberWalk: enabling unconstrained omnidirectional walking through virtual environments. ACM Trans. Appl. Percept. 8 (10.1145/2043603.2043607)

[57] Janeh O, Bruder G, Steinicke F, Gulberti A, Poetter-Nerger M 2018 Analyses of gait parameters of younger and older adults during (non-)isometric virtual walking. IEEE Trans. Vis. Comput. Graph 24, 2663–2674. (10.1109/TVCG.2017.2771520) 29990158

[58] Al Zayer M, MacNeilage P, Folmer E 2018 Virtual locomotion: a survey IEEE Trans. Vis. Comput. Graph. 26, 2315–2334. (10.1109/TVCG.2018.2887379) 30575541

[59] Chance SS, Gaunet F, Beall AC, Loomis JM 1998 Locomotion mode affects the updating of objects encountered during travel: the contribution of vestibular and proprioceptive inputs to path integration. Presence: Teleoperators Virtual Environ. 7, 168–178. (10.1162/105474698565659)

[60] Steinicke F, Bruder G, Kohli L, Jerald J, Hinrichs K 2008 Taxonomy and implementation of redirection techniques for ubiquitous passive haptic feedback. In *Proc. of the 2008 Int. Conf. on Cyberworlds, CW 2008*. Washington, DC: IEEE Computer Society (10.1109/CW.2008.53)

[61] Martelli D, Xia B, Prado A, Agrawal SK 2019 Gait adaptations during overground walking and multidirectional oscillations of the visual field in a virtual reality headset. Gait Posture 67, 251–256. (10.1016/j.gaitpost.2018.10.029) 30388606

[62] Interrante V, Ries B, Lindquist J, Kaeding M, Anderson L 2008 Elucidating factors that can facilitate veridical spatial perception in immersive virtual environments. *Presence: Teleoperators and Virtual Environments* 17, 176–198. (10.1162/pres.17.2.176)

[63] Mohler BJ, Campos JL, Weyel MB, Bülthoff HH 2007 Gait parameters while walking in a head-mounted display virtual environment and the real world. In *Proc. of the 13th Eurographics Symp. on Virtual Environments, Weimar, Germany*, pp. 85–88 (10.2312/PE/VE2007Short/085-088)

[64] Loomis J, Knapp J 2003 Visual Perception of Egocentric Distance in Real and Virtual Environments. In *Virtual and Adaptive Environments*, pp. 21–46. Boca Raton, FL: CRC Press (10.1201/9781410608888.pt1)

[65] Renner RS, Velichkovsky BM, Helmert JR 2013 The perception of egocentric distances in virtual environments – a review. ACM Comput. Surv. 46, 1–40. (10.1145/2543581.2543590)

[66] Steinicke F, Bruder G, Jerald J, Frenz H, Lappe M 2010 Estimation of detection thresholds for redirected walking techniques. IEEE Trans. Vis. Comput. Graph. 16, 17–27. (10.1109/TVCG.2009.62) 19910658

[67] Willemsen P, Colton MB, Creem-Regehr SH, Thompson WB 2004 The effects of head-mounted display mechanics on distance judgments in virtual environments. In *Proc. – 1st Symp. on Applied Perception in Graphics and Visualization, APGV 2004*, pp. 35–38. New York, NY: ACM Press (10.1145/1012551.1012558)

[68] Mohler BJ, Creem-Regehr SH, Thompson WB, Bülthoff HH 2010 The effect of viewing a self-avatar on distance judgments in an HMD-based virtual environment. Presence: Teleoperators Virtual Environ. 19, 230–242. (10.1162/pres.19.3.230)

[69] Bühler MA, Lamontagne A 2018 Circumvention of pedestrians while walking in virtual and physical environments. IEEE Trans. Neural Syst. Rehabil. Eng. 26, 1813–1822. (10.1109/TNSRE.2018.2865907) 30130232

[70] Fink PW, Foo PS, Warren WH 2007 Obstacle avoidance during walking in real and virtual environments. ACM Trans. Appl. Percept. 4, 2 (10.1145/1227134.1227136)

[71] Gérin-Lajoie M, Richards CL, Fung J, McFadyen BJ 2008 Characteristics of personal space during obstacle circumvention in physical and virtual environments. Gait Posture 27, 239–247. (10.1016/j.gaitpost.2007.03.015) 17512201

[72] Moussaïd M, Kapadia M, Thrash T, Sumner RW, Gross M, Helbing D, Hölscher C 2016 Crowd behaviour during high-stress evacuations in an immersive virtual environment. J. R. Soc. Interface 13, 20160414 (10.1098/rsif.2016.0414) 27605166 PMC5046946

[73] Lynch SD, Kulpa R, Meerhoff LA, Pettre J, Cretual A, Olivier AH 2018 Collision avoidance behavior between walkers: global and local motion cues. IEEE Trans. Vis. Comput. Graph 24, 2078–2088. (10.1109/TVCG.2017.2718514) 28650816

[74] Motive:Body. 2019 version 2.1.1. Corvallis, OR: NaturalPoint Inc. https://optitrack.com/downloads/

[75] Autodesk MotionBuilder. 2018 version 2018. San Rafael, CA: Autodesk Inc. https://www.autodesk.com/products/motionbuilder/overview

[76] Adobe Fuse CC. 2014 version 1.2. San Jose, CA: Adobe Inc. https://www.adobe.com/uk/products/fuse.html

[77] Mixamo. 2019 web service. San Francisco, CA: Mixamo Inc. https://www.mixamo.com

[78] Unity. 2019 version 2018.4.0f1. San Francisco, CA: Unity Technologies. https://unity.com/

[79] Polarith AI. 2019 version 1.6. Magdeburg, Germany: Polarith UG. https://polarith.com/ai/

[80] Polarith UG. 2019 Polarith AI documentation. Technical report. Magdeburg, Germany: Polarith UG. http://docs.polarith.com/ai/index.html

[81] Bullimore SR, Burn JF 2004 Distorting limb design for dynamically similar locomotion. Proc. R. Soc. B 271, 285–289. (10.1098/rspb.2003.2611) PMC1691587 15058440

[82] Pheasant ST 1982 Anthropometric estimates for British civilian adults. Ergonomics 25, 993–1001. (10.1080/00140138208925060) 7173163

[83] Bocian M, Macdonald JH, Burn JF 2014 Probabilistic criteria for lateral dynamic stability of bridges under crowd loading. Comput. Struct. 136, 108–119. (10.1016/j.compstruc.2014.02.003)

[84] IEEE. 2009. IEEE Standard for Local and metropolitan area networks—Specific requirements – Part 11. Wireless LAN Medium Access Control (MAC) and Physical Layer (PHY) Specifications – Amendment 5: Enhancements for Higher Throughput. Technical report. IEEE Standards Association (10.1109/IEEESTD.2009.5307322)

[85] NatNet SDK. 2017 version 3.0.1. Corvallis, OR: NaturalPoint Inc. See https://optitrack.com/products/natnet-sdk.

[86] Suiy K, Zhou M, Liu D, Ma M, Pei D, Zhao Y, Li Z, Moscibroda T 2016 Characterizing and improving WiFi latency in large-scale operational networks. In *MobiSys 2016 – Proc. of the 14th Annual Int. Conf. on Mobile Systems, Applications, and Services*, pp. 347–360. New York, NY: Association for Computing Machinery, Inc (10.1145/2906388.2906393)

[87] ARCHICAD. 2019 version 23. Budapest, Hungary: Graphisoft SE. See https://www.graphisoft.com/archicad/.

[88] The MathWorks Inc. 2019 Signal Processing Toolbox™ user's guide (R2019b). Technical report Natick, MA: The MathWorks Inc. See https://uk.mathworks.com/help/pdf_doc/signal/signal.pdf.

[89] MATLAB. 2019 version R2019b. Natick, MA: The MathWorks Inc. See https://uk.mathworks.com/products/matlab.html.

[90] Venkateshan S, Swaminathan P 2014 Numerical Integration. In *Computational Methods in Engineering*, pp. 317–373. Oxford, UK: Elsevier (10.1016/b978-0-12-416702-5.50009-0)

[91] Rosenblum MG, Pikovsky AS, Kurths J 1996 Phase synchronization of chaotic oscillators. Phys. Rev. Lett. 76, 1804–1807. (10.1103/PhysRevLett.76.1804) 10060525

[92] Tass P, Rosenblum MG, Weule J, Kurths J, Volkmann J, Schinitzler A, Freund HJ 1998 Detection of *n*: *m* phase locking from noisy data: application to magnetoencephalography. Phys. Rev. Lett. 81, 3291–3294. (10.1103/PhysRevLett.81.3291)

[93] Welch BL 1947 The generalisation of student’s problems when several different population variances are involved. Biometrika 34, 28–35. (10.1093/biomet/34.1-2.28) 20287819

[94] Wilcoxon F 1946 Individual comparisons of grouped data by ranking methods. J. Econ. Entomol. 39, 269–270. (10.1093/jee/39.2.269) 20983181

[95] IBM SPSS Statistics. 2019 version 26. Armonk, NY: International Business Machines Corporation. See https://www.ibm.com/products/spss-statistics.

[96] Chambers JM, Hastie TJ 1992 Linear models. In *Statistical models in S* (eds JM Chambers, TJ Hastie). Pacific Grove, CA: Wadsworth & Brooks/Cole (10.1201/9780203738535)

[97] Berens P 2009 CircStat: a MATLAB toolbox for circular statistics. J. Stat. Softw. 31, 1–21 (10.18637/jss.v031.i10)

[98] Dang HV, Živanović S 2015 Experimental characterisation of walking locomotion on rigid level surfaces using motion capture system. Eng. Struct. 91, 141–154. (10.1016/j.engstruct.2015.03.003)

[99] Repp BH, Su YH 2013 Sensorimotor synchronization: a review of recent research (2006–2012). Psychonomic Bull. Rev. 20, 403–452. (10.3758/s13423-012-0371-2) 23397235

[100] Rakkolainen I, Raisamo R, Turk M, Hollerer T, Palovuori K 2018 Extreme field-of-view for head-mounted displays. In *3DTV-Conf., Copenhagen, Denmark, 7–9 June*, pp. 1–4. IEEE Computer Society (10.1109/3DTV.2017.8280417)

[101] Howell H 1960 Medical physiology and biophysics. London, UK: Sounders (10.1109/3DTV.2017.8280417)

[102] Alfano PL, Michel GF 1990 Restricting the field of view: perceptual and performance effects. Percept. Mot. Skills 70, 35–45. (10.2466/pms.1990.70.1.35) 2326136

[103] Valkov D, Martens J, Hinrichs K 2016 Evaluation of the effect of a virtual avatar’s representation on distance perception in immersive virtual environments. In *Proc. – IEEE Virtual Reality, Greenville, SC, 19–23 March*. IEEE (10.1109/VR.2016.7504775)

[104] Soczawa-Stronczyk AA, Bocian M 2020 Data from: Gait coordination in overground walking with a virtual reality avatar Dryad Digital Repository. (10.5061/dryad.vx0k6djnr) PMC7428218 32874653

